# Integrative Traditional East Asian Medicine–Based Adjunctive Therapies for Patients With Prostate Cancer Undergoing Standard Cancer Treatment: Protocol for a Systematic Review and Meta-Analysis of Randomized Controlled Trials

**DOI:** 10.2196/94053

**Published:** 2026-07-03

**Authors:** So-Jeong Wi, Dong-Hyeon Kim, Su-Hyon Kim, Sang-Jin Lee, Jong-Sung Park, Seon-Mi Shin, Byung-Cheol Lee, So-Jung Park

**Affiliations:** 1 Department of Korean Internal Medicine School of Korean Medicine Pusan National University Yangsan, Busan Republic of Korea; 2 Department of Korean Internal Medicine Pusan National University Korean Medicine Hospital Yangsan, Gyeongsangnam-do Republic of Korea; 3 Department of Korean Internal Medicine National Clinical Research Center for Korean Medicine Pusan National University Yangsan, Busan Republic of Korea; 4 Department of Internal Korean Medicine College of Korean Medicine Semyung University Jecheon, North Chungcheong Republic of Korea; 5 Department of Clinical Korean Medicine College of Korean Medicine Kyung Hee University Seoul, Seoul Republic of Korea

**Keywords:** Traditional East Asian Medicine, prostate cancer, systematic review, integrative oncology, meta-analysis

## Abstract

**Background:**

Prostate cancer treatment increasingly emphasizes quality-of-life maintenance alongside oncological control. Integrative Traditional East Asian Medicine (TEAM), including traditional Chinese medicine, traditional Korean medicine, and Kampo medicine, has been used as an adjunctive approach for symptom management during cancer treatment. However, evidence regarding its effectiveness and safety across different disease stages remains heterogeneous and has not been comprehensively synthesized.

**Objective:**

This systematic review and meta-analysis aims to evaluate the clinical effectiveness, safety, and quality-of-life benefits of TEAM-based adjunctive therapies combined with standard conventional treatments in patients with prostate cancer.

**Methods:**

Randomized controlled trials will be identified through comprehensive multilingual searches of PubMed, Embase, China National Knowledge Infrastructure, Oriental Medicine Advanced Searching Integrated System, Research Information Sharing Service, and other databases from inception to January 2026. Two reviewers will independently screen studies, extract data, and assess risk of bias using the Cochrane risk of bias 2 tool. Primary outcomes will consist of tumor-related outcomes (prostate-specific antigen levels and Response Evaluation Criteria in Solid Tumors–evaluated tumor response rates) and survival outcomes (overall survival and progression-free survival). Secondary outcomes will include clinical symptoms (International Prostate Symptom Score), pain scores (visual analog scale or numeric rating scale), quality of life, and treatment-related adverse events. Potential herb-drug interactions reported in primary trials will also be systematically assessed. Meta-analyses will be performed using random effects models in RevMan (version 5.4). In cases of substantial heterogeneity (*I*²≥50%), sensitivity and subgroup analyses will be conducted. The certainty of evidence will be assessed with the Grading of Recommendations Assessment, Development, and Evaluation approach.

**Results:**

The study selection process will be illustrated using a PRISMA (Preferred Reporting Items for Systematic Reviews and Meta-Analyses) flow diagram. Where appropriate, pooled effect estimates will be presented using forest plots.

**Conclusions:**

This review will provide comprehensive evidence on the role of TEAM-based adjunctive therapies in prostate cancer care across disease stages and will inform the development of integrative oncology guidelines.

**Trial Registration:**

PROSPERO CRD420251275137; https://www.crd.york.ac.uk/PROSPERO/view/CRD420251275137

**International Registered Report Identifier (IRRID):**

PRR1-10.2196/94053

## Introduction

### Background

Prostate cancer ranks fourth among all cancers and second among male cancers worldwide [1]. In South Korea, it rose from fourth place in 2021 to first among male cancers in 2023, emerging as a major public health concern [2]. Unlike most major cancers—gastric, colorectal, liver, and lung—whose age-standardized incidence rates are projected to decline by 2034, prostate cancer incidence is expected to continue rising [3]. Advances in standard treatments, including hormone therapy, chemotherapy, radiotherapy, and surgery, have improved survival outcomes—with 5-year relative survival reaching 96.9% during 2019 to 2023 in Korea [2]—shifting clinical focus toward symptom management, functional preservation, and quality-of-life maintenance during long-term treatment.

Hormone-sensitive prostate cancer (HSPC) features androgen receptor gene amplification and overexpression, with tumor growth and prostate-specific antigen (PSA) levels highly responsive to circulating androgens [4]. Androgen deprivation therapy (ADT) typically achieves castrate-level testosterone suppression, reducing PSA and tumor burden. Early combination with docetaxel or androgen receptor inhibitors (eg, abiraterone and enzalutamide) delays micrometastatic progression and resistance, improving overall survival [4,5]. Despite these advances, progression to castration-resistant prostate cancer (CRPC), defined by disease advancement despite adequate testosterone suppression, eventually occurs in many patients. In the CRPC stage, treatment strategies include sequential next-generation hormonal agents, docetaxel-based or cabazitaxel-based chemotherapy, radium-223 radionuclide therapy for predominant bone metastases, and poly (ADP-ribose) polymerase inhibitors for patients with breast cancer susceptibility gene or homologous recombination repair mutations [5]. These treatments are associated with cumulative toxicities such as fatigue, myelosuppression, cardiometabolic complications, and neurotoxicity. Thus, in patients with long-term survival potential, symptom management and quality-of-life maintenance are emphasized as critical treatment goals alongside disease control [6].

Risk stratification using PSA level, Gleason grade, and TNM stage classifies prostate cancer into low-risk, intermediate-risk, high-risk, and metastatic categories, guiding tailored therapeutic strategies [5]. Low-risk disease, characterized by slow progression and favorable prognosis, is primarily managed with active surveillance, with selective consideration of local therapies. For intermediate-risk disease, radical prostatectomy or radiotherapy is recommended as primary treatment options, reflecting tumor extent and degree of differentiation. High-risk disease carries an increased risk of local recurrence and distant metastasis, typically necessitating aggressive multidisciplinary treatment, including adjuvant radiotherapy and long-term ADT. Upon progression to CRPC, combination strategies incorporating next-generation hormonal agents, chemotherapy, and targeted therapies become necessary. In metastatic disease, HSPC and CRPC are distinguished based on underlying pathophysiology and molecular characteristics, allowing more refined drug selection and treatment intensity. Prostate cancer most commonly metastasizes to bone (eg, spine, pelvis, and ribs) in advanced stages, leading to skeletal-related events such as pain, fractures, and spinal cord compression that significantly worsen prognosis and quality of life [7].

### Rationale and Objectives

Standard prostate cancer treatments provide oncological benefits but are accompanied by various adverse effects—including hot flashes, night sweats, sexual dysfunction, urinary and bowel disturbances, and fatigue—that impair patients’ daily functioning and quality of life [8-11]. Given that most patients with prostate cancer are aged ≥60 years and have relatively long life expectancy compared with other malignancies [2], long-term symptom management strategies are essential. Furthermore, as disease progresses to CRPC or metastatic stages, treatment sensitivity declines, and standard therapies alone become insufficient for disease control and symptom management, creating a clinical dilemma where survival-prolonging treatments paradoxically lead to cumulative toxicity and functional decline [12]. In this context, there is growing demand for complementary and integrative strategies that alleviate symptom burden and improve systemic well-being without compromising the efficacy of standard anticancer treatments.

Traditional East Asian Medicine (TEAM), including traditional Chinese medicine, traditional Korean medicine, and Kampo medicine, has long been used to alleviate various treatment-related symptoms during cancer care and may serve as a complementary option in settings with limited therapeutic alternatives. Recent studies report clinical benefits from combining herbal medicine and acupuncture with conventional treatments, contributing to the accumulating evidence base for integrative cancer care [13-18]. However, these studies vary widely in intervention types, disease stages, and outcome measures, limiting result consistency. Moreover, no comprehensive synthesis objectively evaluating the effectiveness and safety of TEAM interventions currently exists, leaving a critical gap in standardized clinical consensus. To bridge this gap, a systematic integration of the scattered data is essential to establish a reliable foundation for clinical decision-making. Thus, this protocol systematically reviews the clinical effectiveness and safety of TEAM adjunctive therapies implemented throughout prostate cancer treatment, aiming to provide the necessary scientific evidence for developing evidence-based clinical practice guidelines (CPGs) applicable to clinical practice settings.

## Methods

### Study Registration and Reporting Guidelines

This protocol is registered with PROSPERO (CRD420251275137) and follows the PRISMA-P (Preferred Reporting Items for Systematic Reviews and Meta-Analyses for Protocols) guidelines [19].

### Eligibility Criteria

#### Types of Studies

Only randomized controlled trials evaluating the effectiveness and safety of TEAM-based adjunctive therapies for prostate cancer will be included. Eligible studies must involve human participants. Publications written in English, Chinese, Japanese, or Korean will be considered.

Nonrandomized studies, observational studies, case reports or case series without a control group, crossover trials, preclinical studies, and animal experiments will be excluded.

#### Types of Participants

Participants will include individuals diagnosed with prostate cancer at any disease stage or treatment phase, including those under active surveillance, patients receiving primary treatment, patients in the posttreatment recovery phase, as well as patients with HSPC and CRPC—including both their metastatic forms (metastatic

HSPC [mHSPC] and metastatic CRPC [mCRPC]). No restrictions will be placed on age, ethnicity, disease duration, or geographic location.

#### Types of Interventions and Comparators

The intervention group will receive TEAM-based adjunctive therapies in combination with standard conventional cancer treatments, including surgery, radiotherapy, hormone therapy, or chemotherapy. TEAM-based interventions will include acupuncture, moxibustion, and herbal medicine derived from East Asian traditional medical systems such traditional Chinese medicine, traditional Korean medicine, and Kampo medicine.

Studies in which TEAM-based interventions are not administered as part of the experimental intervention will be excluded. Comparator groups may receive conventional Western medical treatment alone, placebo, or no additional treatment, provided that no TEAM-based interventions are used.

To address potential clinical heterogeneity, prespecified subgroup analyses will be conducted according to intervention modality, including acupuncture-based or moxibustion-based interventions, herbal medicine–based interventions, and combination therapies involving both acupuncture and herbal medicine.

### Outcome Measures

Primary outcomes will consist of 2 main categories: tumor-related outcomes, which specifically focus on changes in PSA levels and tumor response rates evaluated using the Response Evaluation Criteria in Solid Tumors, and survival outcomes, which encompass overall survival and progression-free survival.

Secondary outcomes will include improvement in clinical symptoms (eg, International Prostate Symptom Score for urinary symptoms), pain intensity measured using validated scales (visual analog scale or numeric rating scale), and quality of life assessed using validated measurement tools. Additionally, changes in non-PSA biomarkers, clinical effective rate as defined in the included studies, and treatment-related adverse events evaluated using standardized and validated assessment criteria will be analyzed.

Furthermore, potential herb-drug interactions reported in the included studies will be systematically assessed. Where sufficient data are available, a qualitative or quantitative synthesis of clinically relevant interactions between TEAM products and conventional prostate cancer therapies will be conducted to provide additional safety evidence for clinical practice.

### Search Strategy

A comprehensive literature search will be conducted using both domestic and international electronic databases. Domestic databases will include Oriental Medicine Advanced Searching Integrated System, Research Information Sharing Service, and DBpia. International databases will include PubMed, MEDLINE, Embase, Cochrane Central Register of Controlled Trials, Springer, ScienceDirect, China National Knowledge Infrastructure, CiNii, and Google Scholar.

The search will cover studies published from database inception to January 2026. Search strategies will be developed using a combination of controlled vocabulary terms and free-text keywords related to prostate cancer and TEAM-based interventions. The detailed search strategy for PubMed will be presented in Tables 1-3 and adapted for use in the other databases.

**Table 1:** PubMed database search strategy for herbal medicine.

Number	Search terms
#1	“prostatic neoplasms”[MeSH Terms]
#2	“prostate cancer”[Title/Abstract]
#3	#1 OR #2
#4	“Herbal Medicine”[MeSH Terms]
#5	“drugs, Chinese herbal”[MeSH Terms]
#6	“medicine, traditional”[MeSH Terms]
#7	“medicine, Korean traditional”[MeSH Terms]
#8	“medicine, Chinese traditional”[MeSH Terms]
#9	“Kampo medicine”[MeSH Terms]
#10	“Herbal Medicine”[Title/Abstract] OR “herb*”[Title/Abstract] OR “prescription”[Title/Abstract] OR “decoction”[Title/Abstract] OR “tang”[Title/Abstract] OR “capsule”[Title/Abstract] OR “powder”[Title/Abstract] OR “botanic*”[Title/Abstract]
#11	#4 OR #5 OR #6 OR #7 OR #8 OR #9 OR #10
#12	“randomized controlled trial”[Publication Type]
#13	“controlled clinical trial”[Publication Type]
#14	“randomized”[Title/Abstract]
#15	“RCT”[Title/Abstract]
#16	“random*”[Title/Abstract] AND “allocat*”[Title/Abstract]
#17	“random*”[Title/Abstract] AND “assign*”[Title/Abstract]
#18	#12 OR #13 OR #14 OR #15 OR #16 OR #17
#19	#3 AND #11 AND #18

**Table 2:** PubMed database search strategy for acupuncture.

Number	Search terms
#1	“prostatic neoplasms”[MeSH Terms]
#2	“prostate cancer”[Title/Abstract]
#3	#1 OR #2
#4	“acupuncture”[MeSH Terms] OR “acupuncture therapy”[MeSH Terms]
#5	“auriculotherapy”[MeSH Terms]
#6	“acupuncture”[Title/Abstract] OR “auriculotherapy”[Title/Abstract] OR “electroacupuncture”[Title/Abstract] OR “electro‐ acupuncture”[Title/Abstract]
#7	#4 OR #5 OR #6
#8	“randomized controlled trial”[Publication Type]
#9	“controlled clinical trial”[Publication Type]
#10	“randomized”[Title/Abstract]
#11	“RCT”[Title/Abstract]
#12	“random*”[Title/Abstract] AND “allocat*”[Title/Abstract]
#13	“random*”[Title/Abstract] AND “assign*”[Title/Abstract]
#14	#8 OR #9 OR #10 OR #11 OR #12 OR #13
#15	#3 AND #7 AND #14

**Table 3:** PubMed database search strategy for moxibustion.

Number	Search terms
#1	“prostatic neoplasms”[MeSH Terms]
#2	“prostate cancer”[Title/Abstract]
#3	#1 OR #2
#4	“moxibustion”[MeSH Terms]
#5	“moxibustion”[Title/Abstract] OR “moxa”[Title/Abstract] OR “warm-needle moxibustion”[Title/Abstract] OR “warm needle moxibustion”[Title/Abstract]
#6	#4 OR #5
#7	“randomized controlled trial”[Publication Type]
#8	“controlled clinical trial”[Publication Type]
#9	“randomized”[Title/Abstract]
#10	“RCT”[Title/Abstract]
#11	“random*”[Title/Abstract] AND “allocat*”[Title/Abstract]
#12	“random*”[Title/Abstract] AND “assign*”[Title/Abstract]
#13	#7 OR #8 OR #9 OR #10 OR #11 OR #12
#14	#3 AND #6 AND #13

### Study Selection

All retrieved records will be imported into EndNote (version 20) [20] for reference management and duplicate removal. Two reviewers will independently screen the titles and abstracts of identified studies according to the predefined eligibility criteria. Full-text articles of potentially eligible studies will then be independently assessed for inclusion.

**Figure 1:**
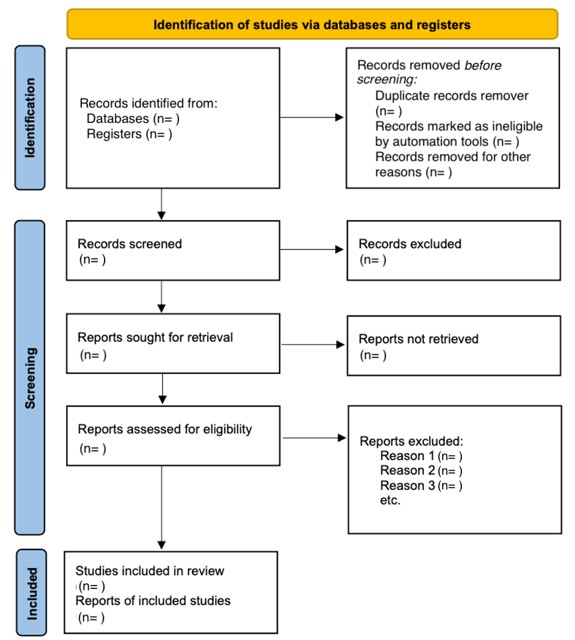
PRISMA (Preferred Reporting Items for Systematic Review and Meta-Analyses) flow diagram of the study selection process.

Any discrepancies between reviewers will be resolved through discussion. If consensus cannot be reached, a third reviewer will be consulted. The study selection process will be illustrated using a PRISMA flow diagram (Figure 1). Reporting will follow the PRISMA-*P* 2015 checklist (Multimedia Appendix 1).

### Data Extraction

Data extraction will be performed independently by at least 2 reviewers using a standardized data extraction form. Extracted data will include study characteristics (first author, publication year, study period), participant characteristics, details of interventions and comparators, outcome measures, duration of follow-up, and reported adverse events. If required data are missing or unclear, attempts will be made to contact the corresponding authors for clarification or additional information.

### Risk-of-Bias Assessment

The risk of bias of included studies will be assessed independently by 2 reviewers using the revised Cochrane risk-of-bias tool for randomized trials [21]. The following domains will be evaluated: bias arising from the randomization process, bias due to deviations from intended interventions, bias due to missing outcome data, bias in measurement of the outcome, and bias in selection of the reported result. Each domain will be judged as having a low risk of bias, high risk of bias, or some concerns. As the revised Cochrane risk-of-bias tool for randomized trials is outcome-specific, assessments will primarily focus on the primary outcomes and key safety outcomes included in the main analyses. Any disagreements will be resolved through discussion or consultation with a third reviewer.

### Data Synthesis and Statistical Analysis

Meta-analyses will be conducted using RevMan software (version 5.4; The Cochrane Collaboration). For dichotomous outcomes, risk ratios with 95% CIs will be calculated. For continuous outcomes measured on the same scale, mean differences with 95% CIs will be used, while standardized mean differences will be applied when different measurement scales are used.

Statistical heterogeneity will be assessed using the Higgins *I*² statistic. Given the anticipated clinical and methodological heterogeneity across studies, including variations in TEAM interventions, treatment regimens, and outcome assessments, random effects models will be used for meta-analyses. Potential sources of heterogeneity will be explored through subgroup and sensitivity analyses. Where sufficient data are available, separate analyses will be conducted according to intervention type, such as herbal medicine, acupuncture, and moxibustion. Where quantitative synthesis is not considered appropriate because of substantial clinical or methodological differences between studies, findings will be summarized narratively.

### Subgroup Analysis

Subgroup analyses will be conducted to explore potential sources of clinical heterogeneity and to evaluate whether treatment effects differ according to disease stage, treatment context, and intervention characteristics. Prespecified subgroup analyses will include disease stage (localized prostate cancer, HSPC, CRPC, metastatic prostate cancer, mHSPC, and mCRPC), conventional treatment background (eg, active surveillance, surgery, radiation therapy, ADT, chemotherapy, or combination therapy), and intervention type (eg, herbal medicine, acupuncture, and moxibustion). Where sufficient data are available, additional analyses will be performed to examine the effectiveness and safety of integrative treatment strategies combining TEAM interventions with conventional therapies.

### Assessment of Reporting Bias

If at least 10 studies are included in a meta-analysis, funnel plots will be generated to assess potential publication bias.

### Sensitivity Analysis

Sensitivity analysis will be conducted to evaluate the reliability of the principal decisions taken during the review process. Factors including small studies, methodological issues, and incomplete data will be assessed throughout the systematic review to facilitate sensitivity analysis.

### Certainty of Evidence

The certainty of evidence for each outcome will be assessed using the GRADE (Grading of Recommendations Assessment, Development, and Evaluation) framework. Evidence quality will be classified as high, moderate, low, or very low based on considerations of study limitations, inconsistency, indirectness, imprecision, and publication bias [22].

### Ethical Considerations

Ethics approval is not required for this systematic review and meta-analysis as it involves no primary data collection or direct patient interventions, but it adheres to high ethical standards in the reporting and handling of data from studies with their ethical clearances. The findings will be disseminated through publication in a peer-reviewed medical journal, presentations at academic conferences relevant to oncology and traditional medicine, distribution via professional networks and societies, and online platforms to ensure wide accessibility and impact in the medical and scientific community.

## Results

This study protocol was registered with PROSPERO on January 6, 2026 (CRD420251275137). At the time of manuscript preparation, pilot testing of the review procedures had been completed. Literature searching, study identification, and screening are underway.

The study selection process will be summarized using a PRISMA flow diagram (Figure 1). Characteristics of included studies will be presented in tabular format. Risk-of-bias assessments and quantitative syntheses will be conducted according to the predefined methodology, and results will be presented using appropriate graphical and statistical approaches, including forest plots where applicable.

The remaining stages of the review, including data extraction, risk-of-bias assessment, evidence synthesis, and manuscript preparation, will be completed sequentially. Completion of the systematic review is anticipated by December 2027.

## Discussion

### Anticipated Findings

Integrating TEAM adjunctive therapies with conventional treatments is anticipated to provide therapeutic benefits by optimizing both objective oncologic parameters and patient-centered clinical outcomes for patients with prostate cancer. We hypothesize that synthesizing scattered clinical data through this systematic approach will demonstrate the synergistic potential of these integrative strategies and effectively resolve existing clinical ambiguities across various disease stages. By establishing a rigorous, consolidated evidence base, these anticipated findings are expected to provide a reliable and definitive foundation for developing evidence-based CPGs in integrative oncology.

### Comparison With Prior Work

TEAM-based adjunctive therapies are gaining interest in integrative oncology for prostate cancer symptom management, particularly regarding serum PSA levels, urinary dysfunction, and pain [13-18]. Although prostate cancer is not explicitly described in classical East Asian medical texts, its clinical manifestations and treatment-related adverse effects have historically been addressed through traditional frameworks emphasizing mass formation, urinary dysfunction, and systemic imbalance [23,24]. These traditional considerations provide a strong theoretical foundation for evaluating TEAM-based interventions through rigorous systematic approaches.

However, existing systematic reviews in this field have largely focused on specific symptoms or individual herbal components, which limit their applicability across the diverse clinical spectrum of prostate cancer [25,26]. This study directly addresses these gaps by implementing a comprehensive and prespecified subgroup analysis based on distinct clinical stages and standard-of-care backgrounds, including localized, advanced (HSPC and CRPC), and metastatic prostate cancer (mHSPC and mCRPC). This structured approach aims to facilitate a more nuanced, evidence-based assessment of the potential role of TEAM-based adjunctive therapies across the entire continuum of prostate cancer care.

### Strengths and Limitations

This review will include studies conducted worldwide; however, most clinical research on traditional medicine has been performed in East Asia, which may result in regional concentration of evidence. To address this limitation, the protocol applies strict eligibility criteria emphasizing randomized study designs, systematic risk-of-bias assessment, and evidence grading. Additionally, stratified quantitative analyses based on intervention type, patient characteristics, treatment duration, and outcome measures are planned to enhance interpretability and transparency.

### Implications

This protocol aligns with ongoing efforts to develop evidence-based CPGs for traditional medicine in oncology. Given the increasing incidence and prolonged survival of patients with prostate cancer, the findings of this study may contribute to the evidence base required for informed clinical decision-making regarding integrative treatment strategies. Ultimately, the results may support the development of multidisciplinary approaches that consider both oncological outcomes and patient-centered care priorities across the continuum of prostate cancer treatment.

### Future Directions

This protocol aligns with ongoing international efforts to establish a robust, evidence-based foundation for integrative oncology. Given the increasing incidence and prolonged survival of patients with prostate cancer, future research stemming from this review will focus on addressing the identified gaps in clinical standardization. Specifically, the insights gained regarding the synergistic potential of combining TEAM with conventional treatments will serve as a critical bridge for designing rigorous, multicenter randomized controlled trials. Ultimately, these future endeavors aim to support the development of comprehensive, multidisciplinary treatment strategies that balance objective oncological outcomes with patient-centered care priorities across the entire continuum of prostate cancer care.

### Dissemination Plan

The findings of this systematic review will be widely disseminated through publication in a peer-reviewed, high-impact journal specializing in integrative medicine or oncology. Additionally, the consolidated results will be presented at major national and international conferences related to traditional medicine and urological oncology. Crucially, the synthesized evidence and clinical insights will be actively shared with clinical guideline development committees to facilitate the formulation of evidence-based CPGs for integrative prostate cancer care. Furthermore, to bridge the gap between research and real-world practice, these outcomes will be used to develop standardized educational and clinical training materials and integrative oncology practitioners, thereby promoting the systematic dissemination of evidence-based protocols in clinical settings.

## Appendix

Multimedia Appendix 1 PRISMA-P 2015 checklist for the systematic review protocol.

## Abbreviations

ADT: androgen deprivation therapy
CPG: clinical practice guideline
CRPC: castration-resistant prostate cancer
GRADE: Grading of Recommendations Assessment, Development, and Evaluation
HSPC: hormone-sensitive prostate cancer
mCRPC: metastatic castration-resistant prostate cancer
mHSPC: metastatic hormone-sensitive prostate cancer
PRISMA: Preferred Reporting Items for Systematic Review and Meta-Analyses
PRISMA-P: Preferred Reporting Items for Systematic Reviews and Meta-Analyses for Protocols
PSA: prostate-specific antigen
TEAM: Traditional East Asian Medicine
